# American Medical Association and Medical Education

**Published:** 1858-04

**Authors:** 


### EDITORIAL.

### AMERICAN MEDICAL ASSOCIATION AND MEDICAL EDUCATION.

It is quite probable that much of the time of the coming annual session of the National Association will be occupied in considering the hackneyed but highly-important subject of medical education.

In the number of our Journal for March, we gave our views in regard to a more systematic and comprehensive course of instruction in the medical colleges; and we will now add a few suggestions in regard to the exaction of a more elevated standard of attainment, and the method by which candidates should be admitted into the profession. That the standard of attainments, both preliminary and medical, required of those who enter the profession is altogether inadequate, is everywhere apparent and acknowledged. The fact is too easily observed to require either argument or elucidation. It is far different, however, with the other branch of the subject, viz: by what method shall we secure the exaction of a standard more elevated and more in accordance with the intrinsic importance and responsibilities of the profession?

Concerning this there is great diversity of opinion. A large part of the profession seem to regard the medical colleges alone as the responsible agents for exacting such a standard as is desirable. This class appears to have controlled the proceedings of the Association at Nashville, and to have procured the appointment of the special committee under the resolutions of Dr. Curry. This committee consisted of Drs. J. R. Wood and John Watson, of N. Y.; Rene LaRoche, of Philadelphia; Geo. R. Grant, of Memphis; and C. B. Nottingham, of Macon; and were instructed to report a "*System* of *Medical Instruction*," which should set forth a "uniform basis upon which our medical institutions shall be organized, * * * * * the *requisite qualifications for graduation*," etc. We have not much confidence that this committee will be able to devise any *system* which will be satisfactory even to those who were most active in procuring its appointment. If it does, we shall be most happy to co-operate in carrying out its recommendations.

Another plan for establishing a proper and uniform standard of attainment, consists in the establishment of a great National University, not for teaching, but for examining candidates and conferring degrees, somewhat after the plan of the present London University. Those who advocate such an institution, would separate from the present colleges the power to confer *degrees* which admit the recipient into the profession, and thereby compel them to rely solely on their merits as institutions for imparting instruction; while the only real avenue into the ranks of the profession would be through the examining board or boards of the National University. Theoretically, this plan is more perfect and more desirable than any other with which we are acquainted; but, unfortunately, we know of no legislative body in our country possessing either the power or the disposition to incorporate such an institution. Congress certainly has no right to charter an university with power to determine the standard of qualifications for the physicians of each State, any more than it has to regulate the qualifications of the lawyers or artizans. And no one will pretend that any State legislature can charter an institution with powers extending beyond the limits of the State creating it. Hence, we do not see how a truly National University, possessing any legal powers, can have an existence in this country. The only practical approximation to this would be a *voluntary* organization, founded on the principles advocated by us in the New York State Medical Society, during the years 1844, '5 and '6, preceding the organization of the American Medical Association. The plan would be as follows, viz: Let the physicians of each State see that their State medical societies are efficiently organized and fully governed by the code of ethics of the National Association. Let the latter designate, fully and clearly, an appropriate and honorable standard of qualifications, both preliminary and medical, for all applicants for recognition as regular members of the profession. Then let each State medical society appoint from its own members an independent board of censors, whose duty it shall be to meet once a year and examine thoroughly all applicants for admission into the ranks of the profession, and to such as are found qualified, diplomas should be granted, by virtue of which they not only become recognized everywhere as physicians, but also as members of the State society, and eligible to election as delegates or members of the National Association. Let the physicians of each State, through their State organization, formally refuse to recognize any as professional brethren except such as either obtain the diploma from some State society, or hold college diplomas obtained anterior to the time of the adoption of the proposed plan; all medical college diplomas issued subsequent to the adoption of this system being regarded in the same light as the degrees A.B., A.M., etc.; that is, as an honorable title, but not of itself constituting the holder a member of one of the most responsible professions in the world.

The adoption of such a plan would effect several important objects.

1st. It would insure greater uniformity and appropriateness in the standard of qualifications, by enabling the National Association, as the common representative of the whole profession, to designate both the standard and the method of carrying it out by the censors of the several State societies

2d. It would greatly increase the importance of the State societies, and the interest which would consequently be felt in their permanency and efficient management. The fees derived from the granting of their diplomas would not only furnish a fund sufficient to pay the board of censors for the time spent in examinations, but would doubtless leave a surplus sufficient to enable each society to offer annually a respectable premium for papers embracing important original investigations.

3d. It would exempt the colleges from the constant jealousy and suspicion engendered by the idea that they are induced to keep the standard of medical education low, for the purpose of increasing the number of their alumni. Being free from the responsibility of actually admitting candidates into the ranks of the profession, they would be left to their legitimate work of *teaching*, and emulating each other only in the extent and perfection of their several courses of instruction.

4th. Finally, it would place the responsibility of admitting new members into the profession where it naturally belongs, namely, with the profession through its own organizations.

We are fully convinced that the adoption of such a plan, coincidently with the more methodical and extensive system of college instruction proposed in our March number, would soon place the cause of medical education in our country on as respectable a footing as it is in any other. It is true, that the whole system would depend upon the voluntary action of the profession, without the coercive force of legal sanction. But what else have we now? Practically, our profession is in no part of this country either legally protected or fostered at the present time. All the distinctions that now exist between the practitioners of legitimate medicine and the advocates of quackery, are made and sustained solely by the force of public sentiment in the profession itself.

And the more perfectly we can develop and embody that sentiment in judicious and important voluntary organizations, the more potent will it become, and the more efficiently will it compel the execution of any plan of education which may be devised and committed to its care.

